# Editorial: Microbiota and metabolites in cancer immunotherapy

**DOI:** 10.3389/fonc.2022.1093941

**Published:** 2022-12-06

**Authors:** Giandomenico Roviello, Martina Catalano

**Affiliations:** Department of Health Sciences, Section of Clinical Pharmacology and Oncology, University of Florence, Florence, Italy

**Keywords:** immunotherapy, cytotoxic T-lymphocyte antigen (CTLA-4), gut microbiota, programmed death/ligand-1, immune checkpoint inhibitors

In the last two decades, immunotherapy has transformed cancer treatment, resulting in unprecedented survival improvement in several advanced and metastatic malignancies. Immune checkpoint inhibitors (ICIs) of cytotoxic T-lymphocyte antigen (CTLA-4) and programmed death/ligand-1 (PD-1/PD-L1) are the main immunotherapeutic tools currently approved, alone or in combination, for the treatment of many tumors (1). However, the downsides of these drugs, including the development of primary or secondary resistance mechanisms to immune-related and occasionally fatal side effects, are well known. Attempts by researchers to identify biomarkers capable of predicting the response to ICIs have been extensive, although not yet completely satisfactory. Continually emerging evidence has confirmed the role of gut microbiota (GM) as immune modulators that can influence the efficacy and toxicity of ICIs (2).

The purpose of this Research Topic is to highlight the evidence regarding the relationship between immunotherapy and GM, and examine the possibility of modulating GM to favor beneficial therapeutic outcomes and improve clinical tolerance to immunotherapy (**Figure 1**).

**Figure 1:**
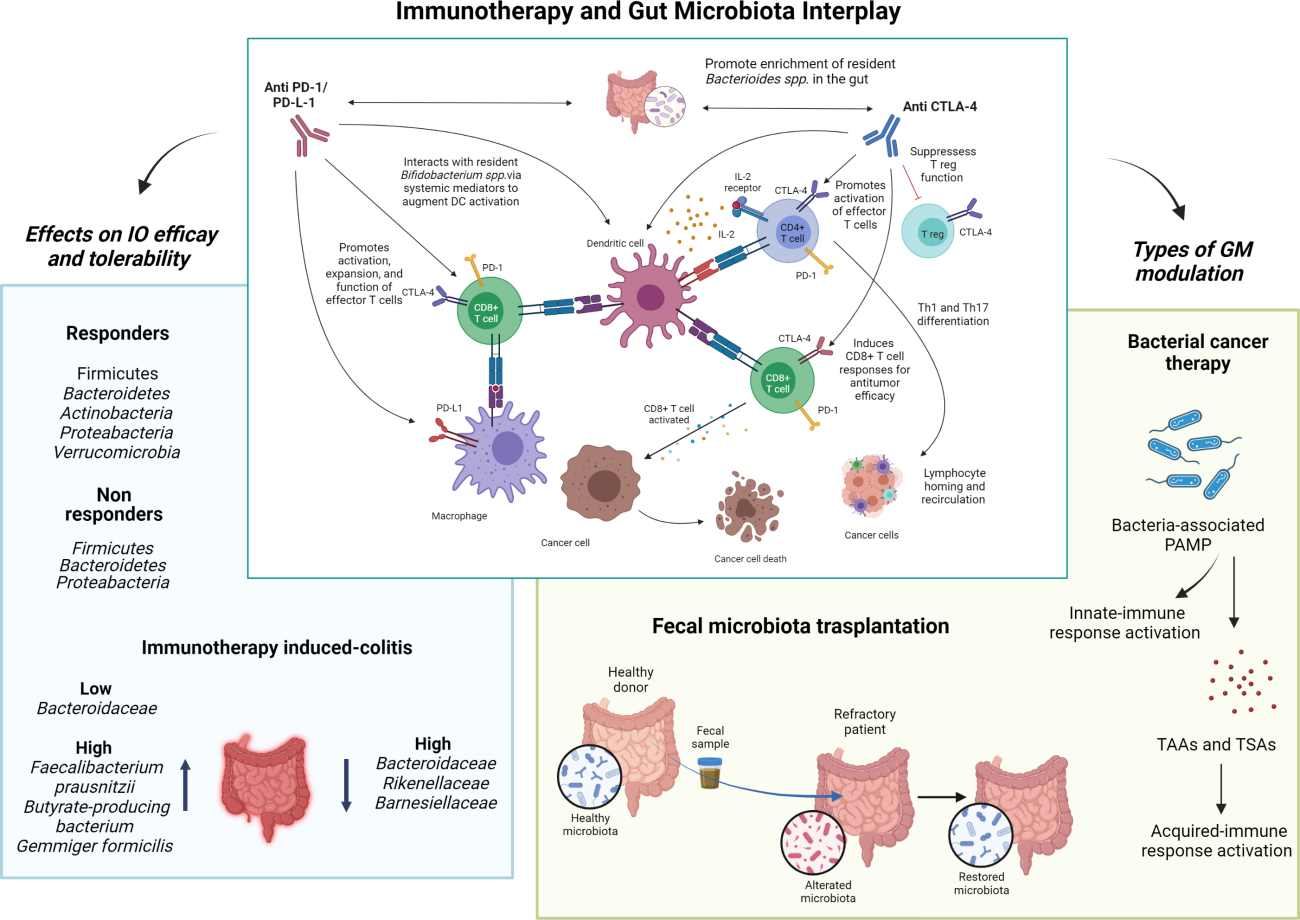
Immunotherapy and gut microbiota interplay. The interaction between gut microbiota and immune checkpoint inhibitors, and the expected consequences on the immune system, is represented in the center. Left, the impact of different microbial species on the efficacy of immune checkpoint inhibitors and immune-related toxicity is shown. Right, the two main gut microbiota modulation strategies discussed in this Research Topic are shown as possible tools for improving the efficacy and toxicity of immunotherapy. I-O, immuno-oncology; GM, gut microbiota; PD-(L)1, programmed death cell-(ligand)1; CTLA-4, cytotoxic T-lymphocytes associated protein-4; PAMP, molecular patterns associated with pathogens; TAAs, tumor-associated antigens; TASs, tumor-specific antigens. Image created using BioRender.com.

Yang et al. conducted a bibliometric analysis of studies evaluating the role of GM in cancer immunotherapy, especially ICIs, and noted that there was a rapid increase in publications in the last 10 years, particularly after 2018. The aim of the study was to understand, through scientometric and visual analysis, the current state of global research regarding interactions between GM and immunotherapy, and anticipate future development directions.

More specifically, Zhou et al. summarized the published research examining the role of microbiota in anti-PD1/PD-L1 therapy, illustrating the underlying mechanisms and providing information on GM manipulation for facilitating PD1/PD-L1 blockade. When considering the literature regarding the synergistic effects of *Bacteroides fragilis* and CTLA-4 blockers, it has been estimated that GM can equally influence the therapeutic effect of anti-PD1/PD-L1. Evidence suggests that gut flora likely functions in modulating PD1/PD-L1 blockade through the translocation of bacteria or dispatch of bacteria-derived molecules to enhance antigenicity and attempt an antitumor immune response. This has been confirmed by observations that some types of microbiotas are explicitly enriched in ICI-responsive patients, whereas others correspond to PD1/PD-L1 non-responders (3–7). With regard to immune-related adverse events (irAEs), although anti-CTLA-4 enrichment of *Bacteroidetes* seems to correlate with a lower risk of mediated colitis, the bacterial species related to the reduced toxicity of anti-PD-1/PD-L1 has not yet been identified (8). Based on the available literature, of all bacteria, those belonging to the *Ruminococacea* family seem to be able to optimize the response to ICI therapy and irAEs (9).

As the effectiveness and toxicity of ICIs seems to be influenced by GM, preclinical data evaluated GM manipulation as a possible technique for enhancing immunotherapy (3, 10, 11). Several approaches, ranging from target interventions, such as bacteriophages and specific bacterial metabolites, to broader strategies, such as antibiotics, pre or probiotics, dietary changes, and fecal microbiota transplantation (FMT), have been tested for their utility in modulating the gut microbiome (12). Of these, FMT is the most direct approach. FMT involves isolating fecal microbiota from stool donated by healthy people and then transplanting them into the patient’s intestine by perfusion or oral administration (13). Xu et al. recently reported that FMT produces a change in patients’ refractory to ICI, making it a promising approach for cancer treatment. Preclinical mouse models demonstrate that FMT can modulate the sensitivity of patients with malignant tumors to ICIs by altering the tumor microenvironment (TME) and enhancing tumor immunogenicity. In patients with immunotherapy-refractory metastatic melanoma, the combination of FMT and anti-PD-1 has recently been shown to be safe, practical, and potentially effective (14). However, in accordance with the authors’ conclusion, although FMT, by remodeling the GM, shows promise in improving the therapeutic efficacy of immunotherapy by reducing side effects, further studies are needed to validate its safety and define the optimal administration regimen for cancer treatment.

By contrast to FMT, bacteria-mediated cancer therapy (BCT) is a method with much older origins in cancer treatment. Starting with Coley’s Toxin in 1983, several strains of therapeutic bacteria have been identified, showing remarkable therapeutic efficacy in preclinical and clinical models (15).

As shown by Luo et al., therapeutic bacteria are able to penetrate and colonize hypoxic areas in tumors, and are sometimes more effective than traditional cancer therapies (16–18). The combination of BCT and chemotherapy has been shown to inhibit tumor growth and prolong survival in preclinical studies, as well as significantly enhance antitumor activity by modulating tumor angiogenesis and host immune response (19, 20). Additionally, in combination with radiotherapy, BCT has been shown to reduce tumor growth in preclinical studies (21), reducing radiotherapy damage in the surrounding tissues (22). Another advantage of administrating live or inactivated bacteriological preparations involves their substantial influence on immune responses. This influence is caused by the action of the molecular patterns associated with pathogens (PAMP), which initiate prompt innate immune responses, characterized by the accumulation of granulocytes and macrophages, as well as an increase in proinflammatory cytokines and chemokines (23–25). Intense bacterial infection and the subsequent innate immune response results in the lysis of tumor cells and the release of tumor-associated and tumor-specific antigens (26), the presence of which in the antigen presenting cell marks the shift to the acquired immune responses. These results provide hope for a possible clinical translation of BCT; however, the use of live bacteria, although highly attenuated, still poses a serious risk of infection and patients must be carefully selected.

## Author contributions

GR had full access to all the data in the study and takes responsibility for the integrity of the data and the accuracy of the data analysis. Study concept and design: GR, MC. Acquisition of data: GR, MC. Analysis and interpretation of data: GR. Drafting of the manuscript: GR. Critical revision of the manuscript for important intellectual content: MC. Statistical analysis: GR. Obtaining funding: None. Administrative, technical, or material support: None Supervision: MC. Financial disclosures: None. Funding/Support and role of the sponsor: None. All authors contributed to the article and approved the submitted version.
